# Supplementary material for: Implementation and Effectiveness of Novel Therapeutic Substances for Advanced Malignant Melanoma in Saxony, Germany, 2010–2020—Cohort Study Based on Administrative Data
Source: Cancers (Basel). 2021 Dec 7;13(24):6150. doi: 10.3390/cancers13246150 (PMC8699477; doi:10.3390/cancers13246150)
Supplement: Supplementary file 1 [file cancers-13-06150-s001.zip › cancers-1484518-supplementary.pdf]

Supplement

Table~S1. Case definitions (metastatic melanoma)

Valid case	Criteria
Incident metastatic melanoma of skin	a) Age ≥18 years<br>b) Continuously insured between 2010 and 2020; deceased persons were included (2012-2020)<br>c) Washout Phase 2010-2011 – individuals with one inpatient or one confirmed outpatient cancer diagnosis (ICD-10-GM C00-C97, except C44) within the washout phase were excluded<br>d) One inpatient record of ICD-10-GM code C43 or at least three outpatient records with C43 within one year in different quarters issued by a medical specialist (not by a general practitioner); first two records with diagnostic modifier 'confirmed', last record without 'suspicion' or 'exclusion'<br>e) In addition the presence of a synchronous or metachronous distant metastasis (one record of ICD-10-GM C78-79 in inpatient or (confirmed) outpatient data at the same time or after first record of melanoma) – for sensitivity all persons with a locoregional metastasis were included (C77)

ICD-10-GM, International Classification of Diseases, 10th Revision, German Modification

Table~S2. Types of therapy and identification codes used

Type of therapy	Sector	Active substance	Used Codes	Coding System
Classical chemotherapeutics				
Chemotherapy	inpatient	Diverse substances	8-542, 8-543, 8-544	OPS
Chemotherapy	outpatient	Dacarbazine	L01AX04	ATC
		Mitobronitol	L01AX01	ATC
		Temozolomide	L01AX03	ATC
		Hydroxycarbamide	L01XX05	ATC
		Estramustin	L01XX11	ATC
		Mitomycin	L01DC03	ATC
		Cyclophosphamide	L01AA01	ATC
		Chlorambucil	L01AA02	ATC
		Melphalan	L01AA03	ATC
		Lomustine	L01AD02	ATC
		Methotrexate	L01BA01	ATC
		Fluorouracil	L01BC02	ATC
		Capecitabine	L01BC06	ATC
	outpatient	Diverse substances	96502	GOP
		Diverse substances	96503	GOP
	outpatient	Diverse substances	9999092	PZN
Interferon	outpatient	Interferon	L03AB	ATC
Novel therapies				
Targeted therapy	inpatient	Vemurafenib	6-006.f	OPS
		Dabrafenib	6-007.5	OPS
		Encorafenib	6-00b.9	OPS
		Binimetinib	6-00b.2	OPS
		Cobimetinib	6-008.c	OPS
		Trametinib	6-009.7	OPS
		Imatinib	6-001.g	OPS
		Dasatinib	6-004.3	OPS
		Nilotinib	6-004.6	OPS
Immune Checkpoint Inhibitor	inpatient	Nivolumab	6-008.m	OPS
		Pembrolizumab	6-009.3	OPS
		Ipilimumab	6-006.j	OPS
		Atezolizumab	6-00a.1	OPS
		Diverse substances	8-547.0	OPS
		Diverse substances	8-547.1	OPS
		Diverse substances	8-547.3	OPS
Targeted therapy	outpatient	Imatinib	L01XE01	ATC

		Dasatinib	L01XE06	ATC
		Nilotinib	L01XE08	ATC
		Vemurafenib	L01XE15	ATC
		Dabrafenib	L01XE23	ATC
		Trametinib	L01XE25	ATC
		Cobimetinib	L01XE38	ATC
		Binimetinib	L01XE41	ATC
		Encorafenib	L01XE46	ATC
Immune Checkpoint Inhibitor	outpatient	Ipilimumab	L01XC11	ATC
		Nivolumab	L01XC17	ATC
		Pembrolizumab	L01XC18	ATC
		Atezolizumab	L01XC32	ATC
	outpatient	Diverse substances	2567478	PZN
	outpatient	Diverse substances	96505	GOP

OPS Operation and Procedure Classification System

ATC Anatomical Therapeutic Chemical Classification System (DIMDI Version 2020)

PZN Pharmazentralnummer - German identification code for pharmaceutical products

GOP Uniform Value Scale - German assessment standard of the outpatient billing data

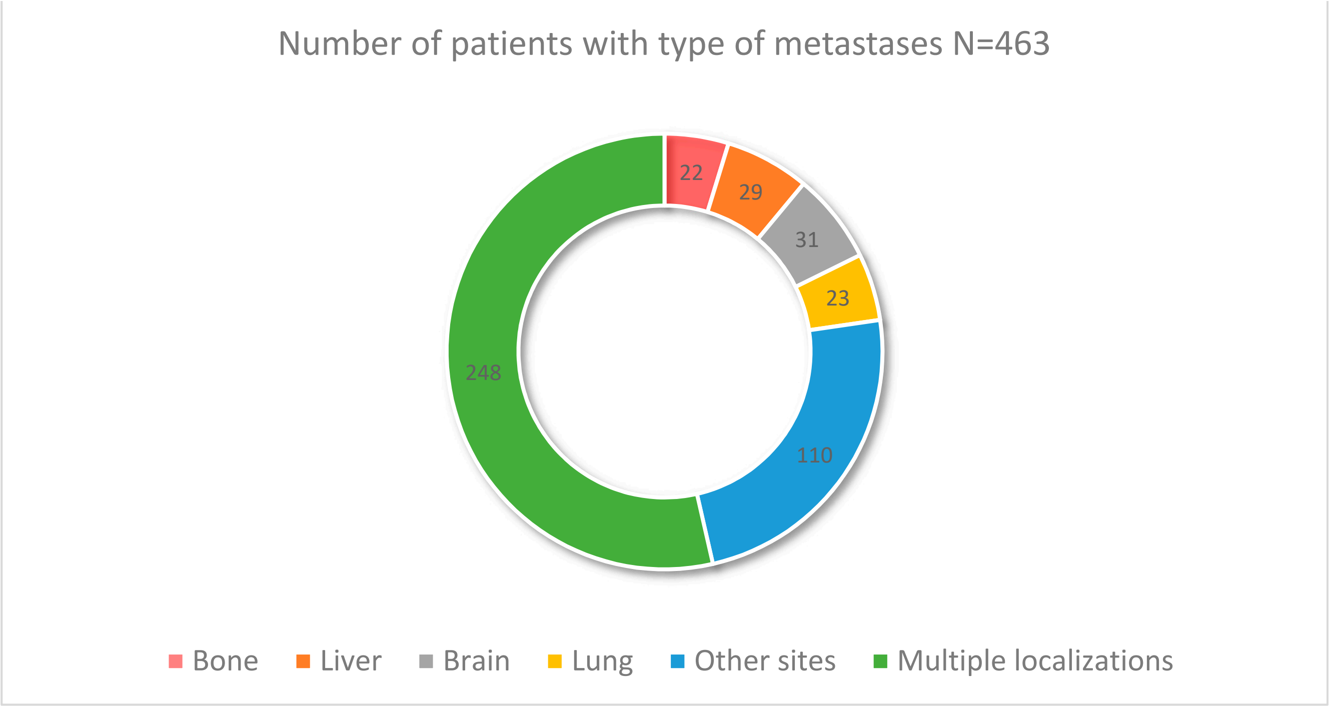

Figure S1. Number of patients with single localizations of metastases in bone, liver, brain, lung, or other sites, and with multiple localizations. About 53% of the patients had metastases in multiple localizations.
